# Mathematical model of fructan biosynthesis and polymer length distribution in plants

**DOI:** 10.1093/aob/mct087

**Published:** 2013-05-03

**Authors:** Susanne Rasmussen, John H. M. Thornley, Anthony J. Parsons, Scott J. Harrison

**Affiliations:** 1 AgResearch Grasslands, Private Bag 11008, Palmerston North, New Zealand; 2 Centre for Nutrition Modelling, Department of Animal & Poultry Science, University of Guelph, Guelph, ON, N1G 2W1, Canada; 3 Institute of Natural Resources, Massey University, Private Bag 11222, Palmerston North, New Zealand; 4 Novo Nordisk Foundation Center for Biosustainability, Danish Technical University, Fremtidsvej 3, Hørsholm, Denmark-2970

**Keywords:** Fructan, *Lolium perenne*, perennial ryegrass, polymer length, model, biosynthesis, mathematics

## Abstract

**Background and Aims:**

There are many unresolved issues concerning the biochemistry of fructan biosynthesis. The aim of this paper is to address some of these by means of modelling mathematically the biochemical processes.

**Methods:**

A model has been constructed for the step-by-step synthesis of fructan polymers. This is run until a steady state is achieved for which a polymer distribution is predicted. It is shown how qualitatively different distributions can be obtained.

**Key Results:**

It is demonstrated how a set of experimental results on polymer distribution can by simulated by a simple parameter adjustments.

**Conclusions:**

Mathematical modelling of fructan biosynthesis can provide a useful tool for helping elucidate the details of the biosynthetic processes.

## INTRODUCTION

*Lolium perenne* (perennial ryegrass) plants containing unusually high concentrations of the fructose polymer fructan (high sugar grasses, HSGs) were obtained in the 1970s by Pollock and Jones (1979). In recent years there has been growing interest in the possible deployment of HSGs in grazed pastures (Turner *et al.*, 2006; Edwards *et al.*, 2007; Parsons *et al.*, 2012) which may increase animal meat and milk production (Lee *et al.*, 2001; Miller *et al.*, 2001), decrease nitrogen (N) deposition to the soil and the generation by the soil of the greenhouse gas (GHG) nitrous oxide (Ellis *et al.*, 2011), and have effects on methane (CH_4_) emanations from grazing animals (Ellis *et al.*, 2012). This could lead to substantial impacts on the environmental footprint of pastoral agricultural systems. For these and other reasons, it is important to understand fructan biosynthesis and degradation, including the dynamics and principal controls of the processes.

Recently, we have developed improved LC-MS (liquid chromatography-mass spectrometry) methods to analyse fructans in ryegrass (Harrison *et al.*, 2009, 2011, 2012), and a series of observations prompted us to reinvestigate the 1-SST/1-FFT hypothesis [1-SST is the sucrose: sucrose 1-fructosyltransferase enzyme, reaction (3) below; 1-FFT is the fructan: fructan 1-fructosyltransferase enzyme, reactions (4) and (5)]. Originally, the 1-SST/1-FFT model was proposed for the inulin-producing species *Helianthus tuberosus* (Edelman and Jefford, 1968), which has subsequently served as the conceptual basis for fructan biosynthesis in other higher plants. The 1-SST/1-FFT paradigm has been criticised, mainly because of discrepancies found between *in vitro* produced fructan oligomer profiles and those present *in planta* (Cairns, 1993; Cairns *et al.*, 2008, and references therein). Fructans in grasses are much more complex than the linear inulin fructans found in, for example, *H. tuberosus* due to the activity of additional fructosyltransferases (FTs) such as 6G-FFT (Lasseur *et al.*, 2006; Hisano *et al.*, 2008) and 6-SFT (Lasseur *et al.*, 2011). These enzymes produce the fructan trimers 6G-kestose and 6-kestose which, when polymerized by FTs, result in the synthesis of levans and the neoseries of inulins and levans. However, from a kinetic point of view all FTs catalyse the same reaction, i.e. the transfer of a fructose from a fructan oligomer to either sucrose or another fructan oligomer and can therefore be summarized into a single reaction equation.

We have constructed a mathematical model of possible pathways of fructan biosynthesis to test a range of assumptions and to compare predictions of the model with observations on fructan concentration and polymer length. Generally, the models are based on: (i) a constant supply of glucose (glc), (ii) the energetically neutral and reversible production of fructose (fru) from glucose [reaction (1)], (iii) the production of sucrose [reaction (2)], (iv) the production of 1-kestose (gf2) by the 1-SST reaction [reaction (3)], (v) the production of fructans of higher degrees of polymerization by successive transfer of fructose from kestose to the growing fructan polymer by FTs [reactions (4) and (5)], (vi) other possible transfers of fructose [reactions (6) and (7)] and (vii) the synthesis and transfer of small fructose polymers [reactions (8)].

Mathematical models of reaction kinetics are used to make predictions of fructan production, including polymer length distributions, which are compared with our own measurements. This may lead to a better understanding of this poorly understood but important area of research.

## MATERIALS AND METHODS

### Plant materials and fructan extraction

*Lolium perenne* seeds from two lines (‘Fennema’, ‘PG113’) were germinated and seedlings transferred to pots containing nutrient-rich potting mix. Plants were grown in controlled environment chambers at two different temperature regimes with a 14-h light and a 10-h dark period. Temperatures were set to either 10 °C constant or to 20 °C during the light and 10 °C during the dark period. Plants were regularly cut back (every 3 weeks) and maintained as described previously (Rasmussen *et al.*, 2009).

Blades (vegetative material above the ligule) were removed after a 3-week regrowth period, immediately frozen and ground in liquid nitrogen and freeze-dried. Ground plant powder (25 mg) was extracted twice in 1 mL 80 % ethanol and subsequently twice in 1 mL of water at 65 °C with constant shaking as described (Rasmussen *et al.*, 2007). Extracts were centrifuged and supernatants combined for MS analysis of fructans. Combined supernatants were brought to dryness under vacuum, reconstituted into 1 mL of water, filtered through a 10-mm filter and transferred into high-performance liquid chromatography (HPLC) glass vials.

### Fructan analysis

Filtrates (5 µL) were injected into the ultra-HPLC system and fructans separated on a Thermo Hypercarb column as described (Harrison *et al.*, 2009). MS analysis was performed using an LTQ ion trap mass spectrometer (Thermo Fisher Scientific, Waltham, MA, USA) with electrospray ionization in negative mode, and data collection over the mass range of 300–4000 allowing the collection of MS data for fructans up to DP (degree of polymerization) 49 (Harrison *et al.*, 2009). Here, we report data corresponding to fructan oligomers in the DP range of DP 3–10 only. As reported previously (Harrison *et al.*, 2011) ions of fructan oligomers with a DP of 3–10 were predominantly singly charged, resulting in mass to charge ratios (*m*/*z*) for the deprotonated molecular ions [M-H]^−^ of the individual DP oligomers of DP3 (=gf2) 503·3, DP4 (=gf3) 665·3, DP5 (=gf4) 827·3, DP6 (=gf5) 989·3, DP7 (=gf6) 1151·3, DP8 (=gf7) 1313·3, DP9 (=gf8) 1475·3 and DP 10 (=gf9) 1637·3.

## FRUCTAN BIOSYNTHESIS: SCHEMES CONSIDERED AND SOME SIMULATIONS

Various schemes are outlined, starting with the simplest. See the Appendix for details of the mathematics, parameterization of rate equations [e.g. eqns (A2), (A14)], and the rate : state equations defining the inputs and outputs for each biochemical species in the model (Thornley and France, 2007, pp. 21, 24).

Throughout, it is assumed that glucose is provided at a constant concentration. Glucose (denoted by glc and also by g) is converted into fructose (denoted by fru and also by f) reversibly according to eqns (A2) and (A14):
(1)
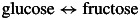



Next glucose and fructose are converted irreversibly into sucrose (glucose–fructose, also denoted by gf1) by means of eqn (A3):
(2)
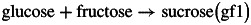



Energy (ATP) is generally required for this reaction (e.g. Thornley and Johnson, 2000, p. 301), although the possible modification of the kinetics by this requirement is not considered here. Any invertase or sucrase action, giving sucrose hydrolysis, is ignored.

### Scheme (a): the basic scheme

Fructan biosynthesis in plants requires multiple, substrate-specific FTs. The currently most widely accepted hypothesis for the biosynthesis of fructans in plants is the 1-SST/1-FFT model proposed by Edelman and Jefford (1968). This hypothesis was originally designed to represent inulin biosynthesis in Jerusalem artichoke (Asteraceae), but is now generally applied to other plant systems as well.

The first step in this reaction sequence is catalysed by the 1-SST enzyme, which facilitates the transfer of a fructose unit from a donor sucrose molecule to the 1 position of the fructose on a sucrose acceptor molecule, forming a β2–1 glycosidic bond and resulting in a 1-kestose molecule (gf2) and glucose (Koops and Jonker, 1996; Van den Ende *et al.*, 1996; Lüscher *et al.*, 2000*a*, *b*; Chalmers *et al.*, 2003). This reaction is
(3)
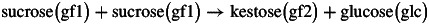



Here, sucrose is denoted by gf1 (glucose-fructose), kestose by gf2 (glucose-fructose-fructose) and the glucose monomer by glc. See eqn (A18) for the rate equation.

The product of this reaction, kestose (gf2), can be used as donor and acceptor for a second fructosyl transfer reaction which is carried out by 1-FFT. This enzyme transfers a fructose unit from the donor 1-kestose molecule to the 1-position of the fructose on an acceptor molecule, forming a β1–2 glycosidic bond, thus extending the fructose chain on the acceptor molecule by one fructose unit; glucose is always terminal. 1-FFT can only use fructans as donor molecules, but not sucrose, while sucrose, fructans and fructose itself [reactions (8)] can act as acceptor molecules (Jeong and Housley, 1992; Koops and Jonker, 1996; Lüscher *et al.*, 1993*a*, *b*; Van den Ende *et al.*, 1996). The reaction for kestose as the acceptor and donor of fructose is [eqn (A25)]:
(4)
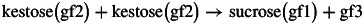

where gf3 denotes the polymer glucose-fructose-fructose-fructose. This reaction can be generalized [eqn(A26)] to
(5)
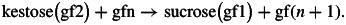



Here *n* is an integer (*n* = 2, 3, … , 9). gfn is a fructan with *n* fructose units attached linearly to glucose. Here we consider polymers up to gf10.

Forage grasses such as *Lolium perenne* (perennial ryegrass) accumulate a mixture of fructan types, namely the inulin and levan series, and the inulin and levan neoseries (e.g. Heldt, 1997, p. 241). Here we just deal with the inulin series, as in (5). These series differ in the glycosidic bonds employed (β2–1 in inulins; β2–6 in levans), and also in the position of glucose in the fructan chain (terminal in the inulin and levan series; internal in the inulin and levan neoseries). The biosynthesis of these fructans requires additional enzyme activities such as 6G-FFT (synthesizes fructans with internal glucose), and 6-SFT (synthesizes levans). The general assumption is that these FTs, like 1-FFT, also transfer single fructose units and can only use fructans (gfn; *n* ≥ 2) as donors, but not sucrose.

**Fig. 1.**
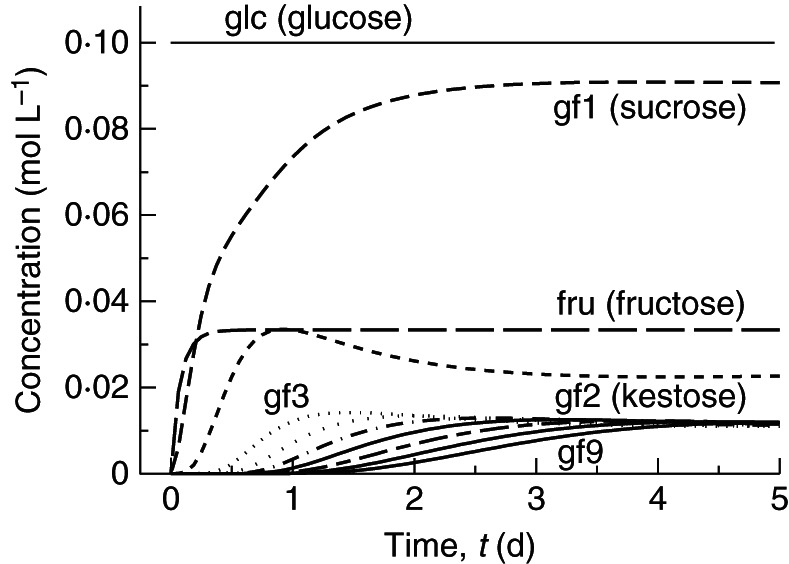
Scheme (a). Time course for scheme (a): the basic scheme, comprising reactions (1)–(5), with default parameters (Table 1). In scheme (a) transfer of a single fructan only occurs from kestose (gf2). Mathematical equations are given in Appendix A [eqns (A2), (A14), (A3), (A18), (A25) and (A26)]. gf*n* (*n* = 3, … , 9) denotes a fructan with *n* fructose units attached to glucose; gf4–gf8 are not labelled due to lack of space.

**Fig. 2.**
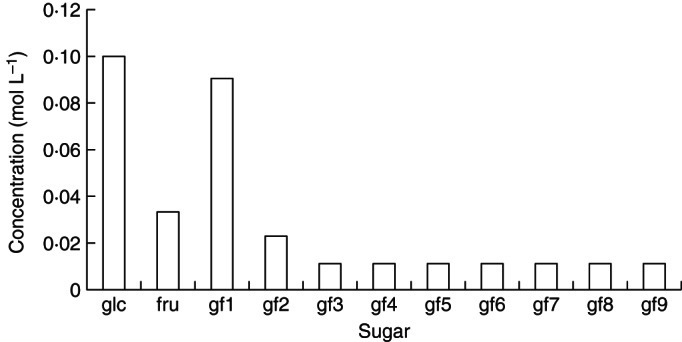
Scheme (a): the steady state. Steady-state concentrations of fructans produced by reactions (1)–(5) as in the basic scheme (a) with default parameterization (Table 1) are shown. Mathematical equations are given in Appendix A [eqns (A2), (A14), (A3), (A18), (A25) and (A26)]. In Scheme (a) transfer of a single fructose only occurs from kestose (gf2). glc denotes glucose; fru, fructose; gf*n*, a fructan with *n* fructose units attached to glucose; gf1 is sucrose; gf2 is kestose.

The basic scheme [Scheme (a)] comprises reactions (1)–(5). FTs use only gf2 (kestose) as a fructose (f) donor; they transfer a single fructose molecule at a time, as in reaction (5) with *n* ≥ 2. Reaction (5) is applied for *n* = 2, 3, … , 9. The reactions proceed, of course, beyond *n* = 9, but we only programmed the problem as far as *n* = 9 as this suffices to describe the essentials of the problem and also our measured data. The time course is given in Fig. 1. Kestose (gf2) and higher polymers (gf3, gf4, … ) all overshoot to a decreasing extent before approaching the steady state. At the steady state (Fig. 2) each fructan polymer (gfn, *n* ≥ 3) reaches the same concentration, which is half that of kestose (gf2). With glucose concentration glc, constant at 0·1 mol L^−1^, the steady-state concentrations are (mol L^−1^): fructose [fru] = 0·0333*; sucrose [gf1] = 0·09; kestose [gf2] = 0·023; and [gfn], *n* = 3, 4, … , 9 = 0·0112 (Fig. 2).

**Table 1.** State variables with initial values, variables, and parameters (concn. = concentration, mol = mole = 1 gram molecule, L = litre = 0·001 m^3^). Default values are given, corresponding to Figs 1 and 2 [Scheme (a), reactions (1)–(5)]. Relevant equation and reaction numbers are indicated

State variable	Definition	Initial values (mol L^−1^)
(a) State variables		
[glc]	Glucose concn. (held constant) (A10)	0·1
[fru]	Fructose concn. (A17)	0
[gf1]	Sucrose concn. (A24)	0
[gf2]	Kestose concn. (A33)	0
[gfn], *n* = 3, 4, 5, 6, … , 9, 10	Fructan with *n* fructose units [(A38), (A44), (A51), (A58), … , (A63), (A67)]	0
[f2]	Fructose dimmer (A70)	0
[f3]	Fructose trimer (A73)	0

Parameter	Definition	Value and units
(b) Parameters		
Michaelis–Menten constants		
*K*_fru_glc_, *K*_glc_fru_	Fructose to/from glucose (1), (A2), (A14)	0·1 mol L^−1^
*K*_glcfru_gf1_	Sucrose synthesis (2), (A3)	0·01 (mol L^−1^)^2^
*K*_gf1gf1_glcgf2_	Kestose synthesis (3), (A18)	0·01 (mol L^−1^)^2^
*K*_gf2gf2_gf1gf3_	gf3 synthesis (4), (A25)	0·01 (mol L^−1^)^2^
*K*_gf2gfn_gf1gf(n+1)_	(5), (A26), *n* = 3, 4, … , 9	0·01 (mol L^−1^)^2^
*K*_gf2f1_gf1f2_, *K*_gf2f2_gf1f3_	Synthesis of fructose dimer and trimer (8), (A27)	0·01 (mol L^−1^)^2^
*K*_gf2f3_gf5_	Last of reactions (8), (A28)	0·01 (mol L^−1^)^2^
*K*_gf3gfn_gf2gf(n+1)_	Reaction (6), (A35), *n* = 6, 7, … , 9	0·01 (mol L^−1^)^2^
Maximum velocities of reactions (mol reactant L^−1^ d^−1^)		
*v*_fru_glc_, *v*_glc_fru_	Fructose to/from glucose (1), (A2), (A14)	1
*v*_glcfru_gf1_	Sucrose synthesis (2), (A3)	1
*v*_gf1gf1_glcgf2_	Kestose synthesis (3), (A18)	1
*v*_gf2gf2_gf1gf3_	gf3 synthesis (4), (A25)	1
*v*_gf2gfn_gf1gf(n+1)_	(5), (A26), *n* = 3, 4, … , 9	1
*v*_gf2f1_gf1f2_, *v*_gf2f2_gf1f3_	Synthesis of fructose dimer and trimer (8), (A27)	0
*v*_gf2f3_gf5_	Last of reactions (8), (A28)	0
*v*_gf3gfn_gf2gf(n+1)_	Reactions (6), (A35), *n* = 3, 4, … , 9	0

### Scheme (b): extra fructose transfer donor added

**Fig. 3.**
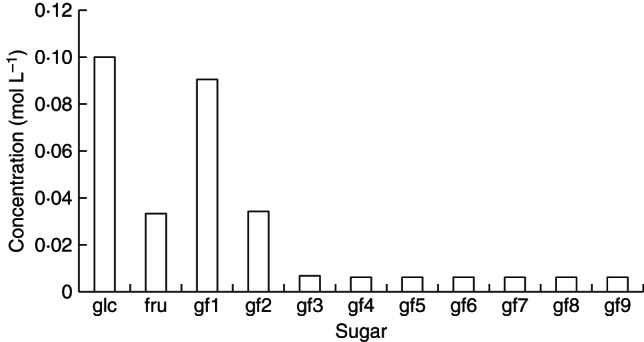
Scheme (b): an extra fructose transfer donor added. Basic scheme (a) [reactions (1)–(5), Figs 1 and 2] is supplemented by the transfer of a single fructose from gf3 (glucose-fructan-fructan-fructan) [reaction (6), eqn (A35)]. Steady-state concentrations are given. glc denotes glucose; fru, fructose; gf1, sucrose; gf2, kestose; gf*n* (*n* = 3, 4, … , ), a fructan with *n* fructose units attached to glucose. Default parameters (Table 1) are modified with: *v*_3__*n*___2(__*n*__+1)_ = 1, *n* = 3, … , 9.

Modifying (a) above, it is now assumed that FTs can use both gf2 (kestose) [as in the basic scheme (a)] but also gf3 (glucose-fructose-fructose-fructose) as a fructose donor with transfer of a single fructose molecule. Thus, in addition to reactions (1)–(5), the reaction [eqn (A35)]
(6)
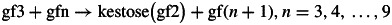

is included. Comparing Fig. 3 with Fig. 2, the steady-state concentrations of individual gfn (*n* ≥ 3) are now, relative to gf2 which is higher, much lower. With glucose concentration ([glc]) constant at 0·1 mol L^−1^, the steady-state concentrations are (mol L^−1^): fructose [fru] = 0·0333*; sucrose [gf1] = 0·09; kestose [gf2] = 0·034; [gf3] = 0·0068; and [gfn] (*n* = 4–9) = 0·0062 (see Fig. 3).

### Scheme (c): effect of a fast reaction

**Fig. 4.**
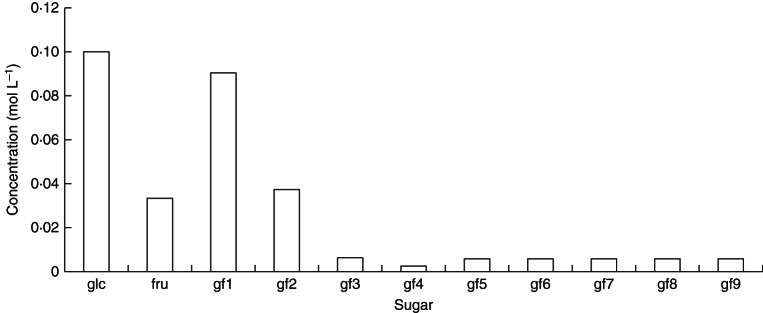
Scheme (c): effect of a fast reaction. Steady-state concentrations of fructans are shown. Parameterization is as in Scheme (b) where *v*_3__*n*___2(__*n*__+1)_ = 1, *n* = 3, … , 9 in reaction (6) [Fig. 3, reactions (1)–(6), eqns (A2), (A14), (A3), (A18), (A25), (A26) and (A35)]. However, now a high maximal velocity is assigned to reaction (7), namely *v*_34_25_ = 10 [eqns (A35) with *n* = 4]. glc denotes glucose; fru, fructose; gf1, sucrose; gf2, kestose; gf*n*, a fructan with *n* fructose units attached to glucose.

This scheme simulates the production of fructans as described under scheme (b) (Fig. 3), but where one of the reactions of reaction (6), namely that with *n* = 4:
(7)
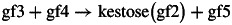

has its maximum velocity (*v*_34_25_) set to a high value, that is *v*_34_25_ = 10 [eqns (A35) with *n* = 4]. The result of this tenfold increase is a strong depression of the concentration of acceptor gf4 (Fig. 4). That is, a gap is generated in the distribution of fructans according to degree of polymerization. With constant glucose ([glc]) concentration of 0·1 mol L^−1^, the steady-state concentrations (mol L^−1^) of the other variables are: fructose [fru] = 0·0333*; sucrose [gf1] = 0·90; kestose [gf2] = 0·037; [gf3] = 0·0063; [gf4] = 0·0025; [gfn] = 0·0058, *n* = 5, … , 9 (Fig. 4).

### Scheme (d): fructose transfers to fructose polymers

The occurrence of oligomeric carbohydrates containing exclusively fructose units (without any glucose) has been described in early studies on the carbohydrate composition of monocotyledons (see Archbold, 1940, and references therein). We therefore added an additional scheme (d), with putative transferase enzymes catalysing the following reactions:
(8)
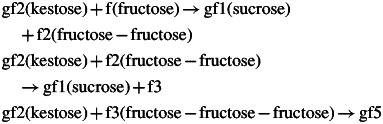



In the first two reactions, kestose (gf2) donates a single fructose, which can be accepted by fructose (f) or by f2 [eqns (A27)], yielding sucrose as a product. In the last reaction, an enzyme catalyses transfer of gf2 (kestose) to f3 [eqns (A28)], giving a gf5 molecule with no other product.

The basic reaction set of scheme (a) [reactions (1)–(5), eqns (A2), (A14), (A3), (A18), (A25) and (A26)] is applied as usual but one of the reactions, namely reaction (4) catalysed by 1-FFT, is made zero by setting the velocity for this reaction to zero: *v*_22_13_ = 0 [eqn (A25)]. There is no production of gf3 or therefore gf4 [reaction (5) with *n* = 3].

**Fig. 5.**
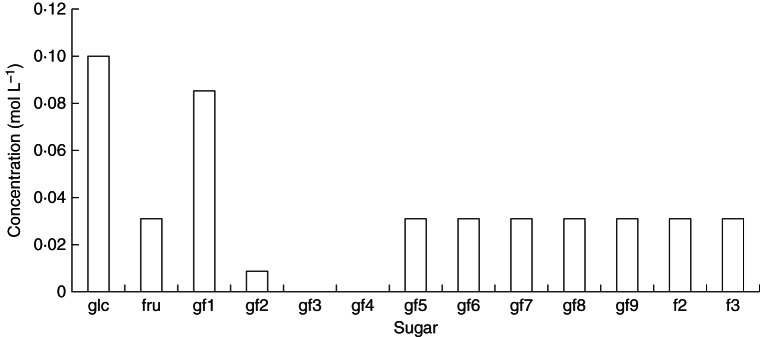
Scheme (d): fructose transfers to fructose polymers allowed. Steady-state concentrations of fructans are shown. The basic scheme (a) is assumed [reactions (1)–(5)] but with reaction (4) (gf2 + gf2 → gf1 + gf3) switched off, *v*_22_13_ = 0 [eqn (A25)]. Additional reactions (8) are added for transfer of fructose (f) from gf2 (kestose) to f (fructose) and to f2 (fructose-fructose) [eqns (A27)] and also for the gf2 (kestose) + f3 → gf5 polymerization [3rd of reactions (8), (A28)]. Default parameters (Table 1) are modified with *v*_2f1_1f2_ = 1, *v*_2f2_1f3_ = 1 and *v*_2f3_5_ = 1 [(8), (A27), eqns (A28)]. glc denotes glucose; fru, fructose; gf1, sucrose; gf2, kestose; gf*n*, a fructan with *n* fructose units attached to glucose; f2, difructose; f3, trifructose.

At the steady state, concentrations of kestose (gf2) were very low and gf3 and gf4 were not produced at all, making a hole in the polymer distribution. Fructans with gfn (*n* = 5–9) and f2 and f3 reached high concentrations, comparable to those of sucrose (gf1). With constant glucose concentration, [glc] of 0·1 mol L^−1^, steady-state concentrations of the other variables are (mol L^−1^): fructose [fru] = 0·031; sucrose [gf1] = 0·085; kestose [gf2] = 0·0087; [gf3] = 0; [gf4] = 0; [gf5–9] = 0·031; [f2] = 0·031; [f3] = 0·031 (Fig. 5).

### Scheme (e): comparison with data

As described above we grew ten genotypes from two *L. perenne* lines, the European cultivar ‘Fennema’ (F) and an experimental breeding line ‘PG113’ (P) in two temperature regimes (one at constant 10 °C – 10/10, the other at 20 °C during the light and 10 °C during the dark period – 20/10). In blades (tissue above the ligule) harvested after 21 d of re-growth after defoliation ‘PG113’ accumulated significantly higher concentrations of total water-soluble carbohydrates (mg g^−1^ d. wt) compared with ‘Fennema’ in both the 10/10 (means P = 322·2, F = 236·3; *P* < 0·0001) and the 20/10 (means P = 341·1, F = 258·6; *P* < 0·0001) treatments (Rasmussen *et al.*, 2009). We chose 21 d post-defoliation blade material as it had been shown previously that at this time the expression and activity of exohydrolases is negligible and that fructan polymerization is prevalent in these tissues (Morvan *et al.*, 1997; Lasseur *et al.*, 2007; Lee *et al.*, 2010; Tamura *et al.*, 2011).

**Fig. 6.**
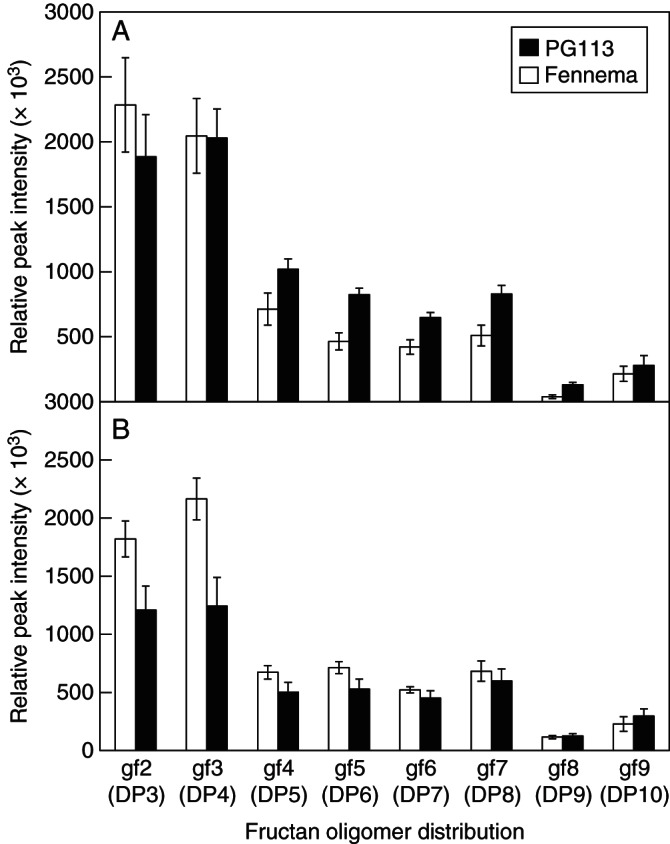
Distribution of relative intensities (peak areas) of the sum of *m*/*z* signals corresponding to fructan oligomers DP3–10 (e.g. gf2/DP3 denotes glucose-fructose-fructose etc.) analysed by LC-MS. The data apply to the leaf blades of *L. perenne* ‘Fennema’ and ‘PG113’ (as indicated) harvested 21 d after defoliation, grown at 14 h light and 10 h dark temperatures of (A) 10/10 °C and (B) 20/10 °C. Vertical bars represent ± s.e.

To determine the distribution of fructan oligomers with different DP, extracts of the above material were analysed by ion trap MS (Harrison *et al.*, 2009). For most of the individual fructan oligomers, we detected several peaks corresponding to the *m*/*z* of the individual fructan oligomers. Specifically, we detected 5 peaks with the *m*/z of 503·3, two of which represent the non-fructan sucrosyl-galactosides raffinose and loliose (Amiard *et al.*, 2003), the other three the fructan oligomers 1-kestose, 6-kestose and 6G-kestose (Harrison *et al.*, 2012). The latter three are represented in Fig. 6 as the sum of fructan trimers [gf2 (DP3)]. Four peaks each with *m*/*z* ratios of 665·3, 827·3, 989·3 and 1151·3 were detected, representing inulin and levan (neo)series fructan tetra-, penta-, hexa- and heptamers, respectively. The sums of peak intensities of each *m*/*z* are shown as gf3 (DP4), gf4 (DP5), gf5 (DP6) and gf6 (DP7) in Fig. 6. Three peaks with *m*/*z* 1313·3 [sum = gf7 (DP8)], one peak with *m*/*z* 1475·3 [gf8 (DP9)] and two peaks with *m*/*z* 1637·3 [sum = gf9 (DP10)] were also detected (Fig. 6).

As can be seen in Fig. 6, the pattern of fructan oligomer distribution in blades followed a comparable trend for both *L. perenne* lines and at both temperature regimes (Fig. 6). The highest relative intensities of DP3–10 fructans in blades were detected for tri- and tetrameric oligomers (DP3 and DP4), followed by approx. 4-fold lower intensities of *m*/*z* signals corresponding to DP5–8 fructan oligomers. The lowest intensities were detected for DP9 oligomers with slightly higher concentrations of DP10 fructans.

**Fig. 7.**
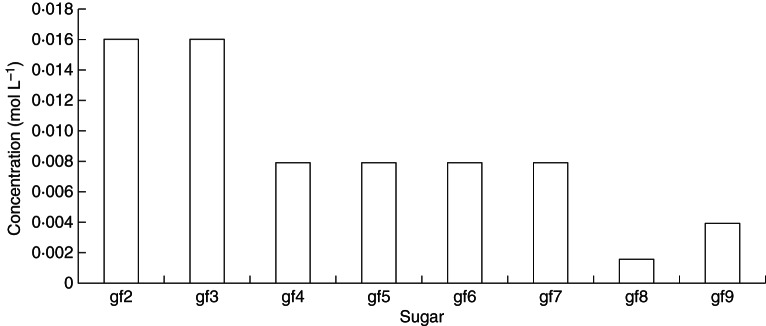
Scheme (e): comparison with data (shown in Fig. 6). Scheme (a) is applied [reactions (1)–(5); eqns (A2), (A14), (A3), (A18), (A25) and (A26)]. Only non-zero default parameters are altered. Changes to the default parameters (Table 1) are: *v*_22_13_ = 2, *v*_24_15_ = 2, *v*_25_16_ = 2, *v*_26_17_ = 2, *v*_27_18_ = 2, *v*_28_19_ = 10, *v*_29_110_ = 4. Steady-state concentrations are: [gf2] = [gf3] = 0·016, [gf4] = [gf5] = [gf6] = [gf7] = 0·0079, [gf8] = 0·0016, [gf9] = 0·0039 mol L^−1^. gf*n* denotes a fructan with *n* fructose units attached linearly to glucose.

Parameters of our model have been adjusted to see if it is possible to obtain agreement with our recent measurements. In fact, it is very easy to obtain such agreement, as can be seen when Fig. 7 (model predictions) is compared with observations in Fig. 6. In Fig. 7, minimal parameter changes have been made to the simplest model scheme with default parameters [(Table 1; scheme (a); Fig. 2; reactions (1)–(5), eqns (A2), (A14), (A3), (A18), (A25) and (A26)]. The velocity parameters alone were adjusted to give the results shown in Fig. 6, focusing on the relative amounts of fructan trimers (gf2, glucose-fructose-fructose) through to gf9, a glucose with nine fructoses attached to glucose, and ignoring the concentrations of glucose, fructose and sucrose. We do not believe our particular parameterization (Fig. 7) is unique and there are likely to be other sets of parameter values that would do equally well.

## DISCUSSION

This paper presents a ‘proof-of-concept’ position, demonstrating that constructing and simulating specific biosynthetic schemes for fructan biosynthesis may significantly assist in understanding these processes. The mathematical analysis and computations are straightforward and, arguably, such approaches could be part of the standard armoury of techniques which is brought to bear on these problems.

More particularly, Figs 2–5 illustrate, using simple assumptions, the range of responses of polymer length distribution which can be obtained. Note also that the simulations presented here deal with fructan biosynthesis alone, without the possibly conflicting and confounding effects from turning on fructan degradation processes (a topic that could be given a similar treatment).

Figure 7 demonstrates that our scheme is able to ‘explain’ measured data (presented in Fig. 6), although many more measurements would be needed to support or refute any detailed scheme proposed with its mathematical and numerical assumptions. The only parameter change we introduced is a 5-fold increase in velocity of the reaction leading to the synthesis of gf8 and a 2-fold increase of the velocity leading to gf9. These new parameters resulted in high concentrations of tri- and tetrameric fructan oligomers, relatively lower concentrations of penta-, hexa-, hepta- and octamers, a very low concentration of gf8 (DP9) and slightly higher concentrations of gf9 (DP10). One way of achieving this higher velocity in plants is the activity of an additional FT with a high affinity for higher DP fructans. Such FTs have been described for *Echinops ritro* (Van den Ende *et al.*, 2006) and *Phleum pratense* (Tamura *et al.*, 2009). To date no such high DP FT has been isolated from *L. perenne* and our model and data might indicate that the identification of such an enzyme would help to explain the observed differences of *in vitro* synthesized fructan profiles obtained by assaying known native *L. perenne* FTs compared with plant fructan profiles (Cairns *et al.*, 2008).

In view of the possible importance of the high-sugar phenotype in grassland productivity, in mitigating GHG emissions and in possibly promoting carbon sequestration, we believe that a detailed mechanistic understanding given by analyses along the lines presented here may enable us to better understand the contributions of the high-sugar traits to grassland ecosystems. A worthwhile aim is to represent such characteristics realistically in grassland ecosystem models (e.g. Thornley, 1998).

In conclusion, it has been demonstrated that mathematical analysis and simulation, based on well-established biochemical kinetics and calculus, is able to play a role in furthering our knowledge of fructan biochemistry, with its potential importance to the environmental and economic consequences of grassland agriculture.

## APPENDIX. MATHEMATICAL DETAILS AND PARAMETERIZATION OF THE MODEL

The equations given below were programmed in ACSL (Advanced Continuous Simulation Language, Aegis Research, Huntsville, AL, USA; version 11·8·4), an ordinary differential equation solver, using fourth-order Runge–Kutta integration and a time step of 0·03125 (=1/32) d. The time unit of the model is days (d). There were no problems in model implementation.

We step through the biochemical state variables pool by pool, giving the outputs from, the inputs to, and the differential equation of each pool. A constant volume of reactant, 1 litre (L), is assumed. A molar concentration is 1 gram molecule (mol) per litre (mol L^−1^), and a flux of 1 mol glucose L^−1^ d^−1^ is 1 gram molecule of glucose per litre per day.

Fluxes are calculated as outputs *O* from a substrate pool, using a mechanistic equation, usually the Michaelis–Menten equation or something similar for bisubstrate reactions (Thornley and France, 2007, pp. 107–109, 113–114). If there is no substrate in a pool, there can be no output. Apart from the system input [eqn (A8)], inputs *I* are the consequence of outputs *O* from other pools, sometimes applying a simple stoichiometry, e.g. [eqns (A5), (A6)].

For a single substrate (*x*) single product (*y*) reaction, fluxes are calculated and designated as outputs (*O*_*x*_*y*_) from substrate pool *x* towards a product pool *y* with units of mol *x* per litre per day. Inputs to pool *y* from the *x* → *y* reaction are designated *I*_*x*_*y*_ (mol *y* L^−1^ d^−1^). Where two substrates (*x* and *y*) are required giving two products (*z* and *u*), the output flux is designated *O*_*xy*_*zu*_ and is calculated as an output from one of the substrate pools (say *x*) which requires a stoichiometrically related flux from the second substrate pool (*y*). The input flux *I*_*xy*_*zu*_ is in units of mol *u* L^−1^ d^−1^ or mol *z* L^−1^ d^−1^ into the *u* and *z* pools. Parameter values are given where they are introduced and are listed in Table 1 for reference.

### Glucose (glc) pool

Synthesis of fructans is driven by maintaining a constant concentration of glucose [glc], namely

(A1)

#### Outputs

The first output flux from the glucose pool is to fructose [the forward reaction (1)] (mol glucose L^−1^ d^−1^)

(A2)

This is a traditional Michaelis–Menten (MM) equation. The parameters are: maximum velocity *v*_glu_fru_ achieved at high values of [glc]; and an MM constant *K*_glc_fru_ giving the half-maximal-velocity glucose concentration [glc].

The second output flux is due to the two-substrate reaction (2) for sucrose synthesis (denoted by gf1, for glucose-fructose):

(A3)

Again, the parameters are: a maximum velocity, *v*_…_ and a MM constant, *K*_… ,_ this time for a two-substrate reaction. This output flux is removed from the fructose pool [eqns (A15)] as well as equally from the glucose pool and it is put into the sucrose (gf1) pool [eqn (A19)].

Total output from the glucose pool is (mol glucose L^−1^ d^−1^)

(A4)

#### Inputs

There is an input from the fructose → glucose reaction (1):

(A5)

This is calculated as an output from the fructose pool in eqn (A14).

There is a second input to the glucose pool from the 1-SST reaction for kestose synthesis (3). This is calculated as (mol glucose L^−1^ d^−1^)

(A6)

*O*_gf1gf1_gf2glc_ is an output from the sucrose pool (gf1) [eqn (A18)] below.

Adding these two input fluxes gives a total input flux of

(A7)

The glucose concentration is (by assumption) held constant. This is achieved by adding an external (ext) input flux of

(A8)

Total input flux is

(A9)

#### Differential equation

The rate of change of glucose concentration is

(A10)

In view of eqn (A9), d[glc]/d*t* is zero, and the glucose concentration remains at its initial value of 0·1 mol L^−1^.

Note that the total input to the system is (mol glucose L^−1^ d^−1^)

(A11)

There are no outputs from the system so the rate of change of the whole system variable (*S*_*y*s_ mol glucose L^−1^ d^−1^) is

(A12)

A useful check on the formulation and programming of the problem is also to calculate the rate of change of the whole system by summing its parts:

(A13)

Equations (A12) and (A13) should be identically equal at all times.

### Fructose (fru) pool

#### Outputs

The first output is to glucose via reaction (1) in the reverse direction:

(A14)

This is the mirror image of eqn (A2). The second output is to sucrose synthesis, *O*_glcfru_gf1_, in eqn (A3) above. The third output, *O*_gf2f1_gf1f2_, is for the synthesis of the fructose-fructose dimer (f_2_) [first of reactions (8)], calculated as an output from the kestose (gf2) pool below [first of eqns (A27)]. Total output is

(A15)

#### Inputs

There is a single input to the pool from glucose, with

(A16)

where the output from the glucose pool is calculated in eqn (A2).

#### Differential equation

This is

(A17)

### Sucrose (gf1) pool

#### Outputs

It is assumed that there is a single output from the sucrose pool giving kestose (gf2) synthesis as in reaction (3) (invertase or sucrase activity, promoting sucrose hydrolysis, is ignored). This flux is

(A18)

#### Inputs

The principal input to the pool is due to sucrose synthesis from glucose and fructose, reaction (2), with [eqn (A3)]

(A19)

A second input is from the kestose pool, reaction (4), given by

(A20)

This output from the kestose pool is calculated in eqn (A25). There are also many similar inputs, arising from the outputs of reactions (5) with *n* = 3, 4, … , 9:

(A21)

These outputs from the kestose pool are calculated in eqn (A26).

There are two more inputs, arising from the synthesis of the fructose dimer f2, and the fructose trimer f3, shown in reactions (8):

(A22)

The outputs on the right of these equations are again calculated under the kestose pool, eqns (A27).

Total input to the sucrose pool is

(A23)

#### Differential equation

This is

(A24)

### Kestose pool (gf2)

#### Outputs

There is an output [reaction (4)] giving gf3 synthesis:

(A25)

There are a series of similar outputs from reaction (5) (*n* = 3, 4, … , 9):

(A26)

There can be outputs from kestose of a fructose monomer to a fructose monomer or to a fructose dimer, accomplishing the synthesis of fructose dimers and trimers [reactions (8)] :

(A27)

Note that these reactions are turned off in default giving just the basic reaction scheme [scheme (a), reactions (1)–(5)].

There is one further potential output to be considered, that of the third of reactions (8). This is

(A28)

Total outputs from the gf2 (kestose) pool are

(A29)

#### Inputs

The principal input to the kestose pool is via the 1-SST reaction from sucrose (3) with [see eqn (A18)]

(A30)

There is also a series of inputs from reaction (6) where gf3 can act as a fructose donor [this is outside the basic scheme of reactions (1)–(5)]:

(A31)

The right side outputs are calculated under the gf3 pool below [eqn (A35)]. Total input is

(A32)

#### Differential equation

This is

(A33)

### gf3 pool

#### Outputs

There is an output of reaction type (5) with *n* = 3 giving gf4 synthesis:

(A34)

This is given by eqn (A26) with *n* = 3. There is a series of outputs from reaction (6) (*n* = 3, 4, … , 9):

(A35)

Total output from the gf3 pool is

(A36)

#### Inputs

The only input is via the 1-FFT reaction from kestose (4) with [see eqn (A25)]

(A37)

#### Differential equation

This is

(A38)

### gf4 pool

#### Outputs

There is an output of reaction type (5) and eqn (A26) with *n* = 4 giving gf5 synthesis:

(A39)

There is a second output from reaction (6) and eqn (A35) with *n* = 4:

(A40)

Total output from the gf4 pool is

(A41)

#### Inputs

There are two inputs [with eqns (A26) with *n* = 3 and eqn (A35) with *n* = 3]:

(A42)

Total input is

(A43)

#### Differential equation

This is

(A44)

### gf5 pool

#### Outputs

There is an output of reaction type (5) and eqn (A26) with *n* = 5 to gf6 synthesis:

(A45)

There is a second output from reaction (6) and eqn (A35) with *n* = 5:

(A46)

Total output from the gf5 pool is

(A47)

#### Inputs

The first two inputs are [reaction (5), eqns (A26) with *n* = 4; and reaction (6), eqns (A35) with *n* = 4]:

(A48)

There is a third input from the last of reactions (8) [eqn (A28)]

(A49)

Total input is

(A50)

#### Differential equation

This is

(A51)

### gf6 pool

#### Outputs

There is an output of reaction type (5) [eqn (A26) with *n* = 6] to gf7 synthesis:

(A52)

There is a second output from reaction (6) and eqn (A35) with *n* = 6:

(A53)

Total output from the gf6 pool is

(A54)

#### Inputs

There are two inputs. The first is [reaction (5); eqns (A26) with *n* = 5]:

(A55)

The second is [reaction (6) and eqn (A35) with *n* = 5]:

(A56)

Total input to the gf6 pool is

(A57)

#### Differential equation

This is

(A58)

### gf7 and gf8 pools

The equations are the similar to those for the gf6 pool [eqns (A52) to (A58) ]: for the gf7 pool, change 7 to 8, then 6 to 7 and 5 to 6; for the gf8 pool, change 7 to 9, 6 to 8 and 5 to 7.

### gf9 pool

#### Outputs

There are two outputs:

(A59)

The first is an output of reaction type (5) with *n* = 9 synthesizing gf10 [eqn (A26) ]; the second is from reaction (6) and eqn (A35) with *n* = 9. Total output from the gf9 pool is

(A60)

#### Inputs

The two inputs are [reaction (5), eqns (A26) with *n* = 8; and reaction (6), eqns (A35) with *n* = 8]:

(A61)

Total input is

(A62)

#### Differential equation

This is

(A63)

The gf9 pool is (in the present formulation) the last pool to reach a steady state (Fig. 1). Therefore, the proportional or relative growth rate of gf9, *R*_gf9_ (d^−1^), is calculated to assess the accuracy of the final state. This is

(A64)

### gf10 pool

This is the last pool included in our analysis. It has inputs of [eqns (A26) and (A35) both with *n* = 9]

(A65)

giving a total input of

(A66)

There are no outputs so that gf10 increases linearly in the steady state, when all the pools except gf10 are constant [see eqn (A8) for the total system input, *I*_e*x*t_glc_]. Thus, in the steady state, the equation

(A67)

must be satisfied. This equation provides another useful check on the accuracy of problem formulation and programming.

### f2 pool (fructose dimer)

#### Outputs

The sole output is with the second of reactions (8) given in eqns (A27). Total output from the f2 pool is

(A68)

#### Inputs

There is one input, from the first of reactions (8) and the first of eqns (A27):

(A69)

#### Differential equation

This is

(A70)

### f3 pool (fructose trimer)

#### Outputs

The sole output is with the last of reactions (8) given in eqns (A28). Total output from the f3 pool is

(A71)

#### Inputs

There is one input, from the second of reactions (8) and the third of eqns (A27):

(A72)

#### Differential equation

This is

(A73)
